# Dura mater enhancement on 3T MRI is associated with cortical lesion burden in multiple sclerosis

**DOI:** 10.1007/s00415-026-13781-6

**Published:** 2026-04-06

**Authors:** Robert Zivadinov, Mehak Semy, Alexander Bartnik, Franziska E. Hildesheim, Ashley Tranquille, Dejan Jakimovski, David Hojnacki, Svetlana Eckert, Bianca Weinstock-Guttman, Michael G. Dwyer, Niels Bergsland

**Affiliations:** 1 Buffalo Neuroimaging Analysis Center, Department of Neurology, Jacobs School of Medicine and Biomedical Sciences, University at Buffalo, State University of New York, 77 Goodell Street, Suite 450, Buffalo, NY 14203 USA; 2 Center for Biomedical Imaging, University at Buffalo, State University of New York, Buffalo, NY USA; 3 Lady Davis Institute for Medical Research, Department of Neurology & Neurosurgery, McGill University, Montreal, Canada; 4 Department of Neurology, Jacobs Comprehensive MS Treatment and Research Center, Jacobs School of Medicine and Biomedical Sciences, University at Buffalo, State University of New York, Buffalo, NY USA

**Keywords:** Multiple sclerosis, Dura mater enhancement, Leptomeningeal enhancement, Meningeal perivascular enhancement, Cortical lesions

## Abstract

**Background:**

Post-contrast FLAIR MRI can demonstrate meningeal signal enhancement in people with multiple sclerosis (pwMS), including enhancement along the leptomeninges and dura mater. Although these enhancement patterns have been variably interpreted as reflecting inflammatory processes, their biological specificity and relationship to cortical lesion pathology remain uncertain.

**Objective:**

To evaluate the association between cortical lesion burden and distinct patterns of meningeal enhancement (ME), including dura mater enhancement (DME), leptomeningeal enhancement (LME), and meningeal perivascular enhancement (MPVE) on MRI in pwMS.

**Methods:**

214 pwMS (173 relapsing–remitting, 41 progressive) underwent 3T MRI including 3D FLAIR pre- and post-contrast, and subtraction imaging. ME (LME + DME) and MPVE were visually classified. Cortical lesions were quantified using a validated multi-modal approach incorporating MMCLE, FLAIR-squared, AI-DIR, and T1/T2 ratio maps, with expert verification. Analyses were adjusted for age, sex, disease duration, disease subtype, and disease-modifying treatment, and corrected for multiple comparisons.

**Results:**

142 (66.4%) pwMS showed combined ME/MPVE, 121 (56.5%) ME, including 97 (45.3%) with DME and 48 (22.4%) with LME. Additionally, 46 (21.5%) exhibited MPVE. A higher prevalence and frequency of LME + pwMS were observed in older age groups (*p* = 0.002), whereas no such age-related pattern was found for DME + or MPVE + pwMS. DME + pwMS showed greater cortical lesion number (9.6 vs. 4.9, *p* = 0.003) and volume (374.1 mm^3^ vs. 187.6 mm^3^, *p* = 0.019) compared to DME − pwMS. DME frequency correlated with cortical lesion number (*r* = 0.59, *p* < 0.001) and volume (*r* = 0.56, *p* < 0.001). Stepwise regression identified DME as an independent predictor of cortical lesion number (*β* = 0.45, *p* < 0.001) and volume (*β* = 0.41, *p* < 0.001), explaining 12–15% of variance beyond conventional MRI measures.

**Conclusions:**

DME on 3T MRI is associated with increased cortical lesion burden in pwMS.

**Supplementary Information:**

The online version contains supplementary material available at 10.1007/s00415-026-13781-6.

## Introduction

Leptomeningeal enhancement (LME) on post-contrast MRI has emerged as an in vivo marker of pathological processes occurring at the brain surface in people with multiple sclerosis (pwMS) [1–5]. Direct evidence linking LME detected on MRI to specific inflammatory substrates remains limited. Most supporting literature is based on imaging-only studies, indirect clinicopathological correlations, or small post-mortem series, and similar enhancement patterns have been reported in non-inflammatory neurological conditions, albeit with much lower frequency [1–4, 6, 7]. Accordingly, meningeal enhancement (ME) on MRI should be interpreted as a nonspecific imaging phenomenon reflecting altered meningeal permeability or contrast distribution, rather than a direct surrogate of histologically confirmed inflammation.

LME is detected in approximately 20–50% of pwMS using optimized 1.5T–3T MRI protocols [1, 8–14] and in up to 80–90% at 7T [15–20]. Its presence correlates with higher cortical lesion burden [20], brain atrophy [1, 2, 14], greater disability [2, 10, 19], and a progressive disease phenotype [1, 2, 14, 19]. Collectively, these findings demonstrate consistent associations between ME and cortical pathology, although the underlying biological mechanisms remain incompletely understood.

The meninges are composed of two distinct but contiguous compartments: the leptomeninges (pia and arachnoid mater) and the pachymeninges (dura mater) [21, 22]. The leptomeninges envelop the brain and spinal cord, forming the boundaries of the subarachnoid space where cerebrospinal fluid (CSF) circulates. The dura mater, the outermost fibrous layer, adheres to the inner skull. Both compartments contain rich vascular and lymphatic networks and are populated by resident immune cells, including B cells, T cells, macrophages, and dendritic cells [6, 21–26]. Increasing evidence suggests that the meningeal spaces, through their venous and glymphatic interfaces, act as key gateways for peripheral immune cells entering the central nervous system (CNS) [6, 23–25]. Immune cells may migrate from the dural venous sinuses or meningeal lymphatics through perivascular and subarachnoid channels into the adjacent cortex, establishing chronic inflammatory reservoirs that sustain cortical demyelination and neurodegeneration [1, 2, 19]. Therefore, studying both the leptomeningeal and pachymeningeal compartments together is crucial for understanding how immune infiltration at the CNS borders contributes to cortical injury in pwMS.

While the majority of imaging studies have focused on LME [1, 6, 8, 10–14, 18–20], increasing attention has turned to the pachymeninges, as potential extracerebral sites of inflammatory activity in pwMS [9, 15–17]. Recent MRI investigations have identified the dura mater as a compartment that may exhibit contrast enhancement on MRI at least as frequently, and in some studies with even higher prevalence, than the leptomeninges or meningeal perivascular cortical structures in pwMS [9, 16, 17]. Dura mater enhancement (DME) is observed at both 3T [9, 16] and 7T [15–17] field strengths although its biological significance remains to be clarified. Unlike leptomeningeal vessels, dural vasculature lacks a blood–brain barrier, and DME can be observed in healthy individuals following intravenous contrast administration [7, 16, 27].

Despite emerging evidence, the relationship between cortical lesion burden and distinct patterns of ME remains poorly understood in pwMS. Most prior investigations have concentrated on the presence or prevalence of LME and cortical pathology [14, 18–20], with limited quantification of DME or its interaction with cortical lesion pathology. Moreover, meningeal perivascular enhancement (MPVE) is evidenced as focal contrast enhancement adjacent to meningeal vessels on post-contrast imaging. The biological substrate of this finding is uncertain and cannot be assumed to represent vessel wall inflammation; it may instead reflect contrast accumulation within perivascular or adjacent CSF spaces. Understanding how these enhancement patterns relate to cortical lesion burden may provide complementary insights into distinct routes of inflammatory propagation at the brain surface and its potential link to cortical pathology.

In this study, we investigated the relationship between cortical lesion burden and distinct patterns of ME on 3T MRI. We deliberately avoid attributing specific biological mechanisms to these imaging findings and instead focus on quantifying their associations with cortical lesion number (LN) and lesion volume (LV). This approach builds on prior 7T findings [18], which did not identify a clear association between LME and cortical lesions.

## Methods

### Study population

This was a retrospective study from subjects collected within the Biomarker Understanding for Future Findings and Advancements for Long-term Outcomes in Multiple Sclerosis (BUFFALO-MS) database [28], which is a longitudinal repository of pwMS followed at the Jacobs Comprehensive MS Treatment and Research Center, University at Buffalo, over a 17-year period (2007–2025). The database includes 3863 individuals with MS whose demographic, clinical, and MRI data are compiled biannually and cross-referenced with electronic medical records by trained coordinators and neurologists. Disease status and disability are assessed by MS-trained clinicians using standardized neurological examinations, including the Expanded Disability Status Scale (EDSS).

The inclusion criteria for this study were: (a) diagnosed with MS based on the 2017-revised McDonald criteria [29], (b) having relapsing or progressive MS, (c) availability of demographic (age, sex) and clinical (age at onset, disease duration, EDSS) data at the time of MRI examination, (d) having a brain MRI obtained no longer than 30 days from a clinical examination in absence of relapse, (e) availability of 3T MRI scan with pre- and post-contrast (10 min delay) 3D T2-weighted (w) fluid attenuated inversion recovery (FLAIR), along with 2D T2w, 3D T1w (pre- and post-contrast) and 3D T1w inversion recovery—fast spoiled gradient echo (IR-FSPGR) sequences, (f) MRI data passing quality control procedures to ensure adequate image quality for analysis, and (g) having no other medical conditions associated with brain pathology. A flow diagram summarizing the screening, inclusion, and exclusion of participants from the BUFFALO-MS database for the present study is provided in the Supplement Figure.

A total of 214 consecutive pwMS, consisting of 173 with relapsing–remitting MS (RRMS) and 41 with progressive MS (PMS) fulfilled the inclusion and exclusion criteria, who obtained their MRI examinations in the period between 2017 and 2018, when 3D T2w FLAIR pre- and post-contrast (10 min delay) sequences were used to investigate prevalence of ME in clinical cohort of pwMS in our center [8, 9, 11–13].

Demographic, clinical, and disease-modifying treatment (DMT) data were collected. EDSS was used to determine the level of physical disability. Patients with secondary-progressive (*n* = 32) and primary-progressive (*n* = 9) MS were categorized into one collective group described as progressive MS (PMS).

### Ethics statement

The protocol was approved by the Institutional Review Board (STUDY00005551), and the requirement for informed consent was waived because of its retrospective nature, in accordance with the University at Buffalo guidelines and regulations.

### MRI acquisition and analyses

Subjects underwent MRI examination on a 3T GE Signa Excite HD 23.0 scanner (GE, Milwaukee, WI) with a 16-channel head and neck coil. A 3D T2w FLAIR sequence as well as a 3D T1w spin echo were acquired before and 10 min after (for 3D T1w, 5 min after) a single dose of intravenous 0.1 mmol/kg Gad injection. A 3D T1w IR-FSPGR was also acquired without contrast. Details of the MRI sequence acquisition protocol are provided in the Supplement Table 1.

All image analyses were performed in a blinded manner without knowledge of the subjects’ demographic details or clinical conditions.

### Lesion analysis

T2-LV, T1-LV, and contrast-enhancing (CE)-LV and LN were obtained using a semi-automated contouring/thresholding technique using Java Image Manipulation (JIM) (version 6.0; Xinapse System; https://www.xinapse.com) [30].

**Fig. 1:**
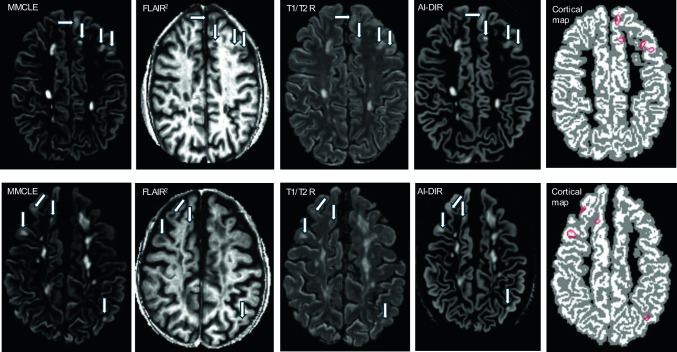
Representative example of the images and layout used for simultaneous cortical lesion detection and/or review. Example of well-visualized cortical lesions are highlighted (arrows). From left to right: multi-modal cortical lesion enhanced (MMCLE) image, a combination of FLAIR-squared (^2^) and artificial intelligence-double inversion recovery (AI-DIR); FLAIR^2^, a combination of FLAIR and T2-WI; T1/T2 ratio (*R*) image; AI-DIR, synthesized based on a deep neural network from the FLAIR, T1, PD, and T2 images and cortical map from FastSurfer (actual cortex shown in white, cortical lesions shown in red circles, voxels within 2 mm of cortex, including from above and below, in gray). The top row shows cortical lesions in a 41 relapsing–remitting male with 28 cortical lesions and 1281 mm^3^ of cortical lesion volume who presented with 3 foci of dura mater enhancement. The bottom row shows cortical lesions in a 52 secondary-progressive female with 54 cortical lesions and 3080 mm^3^ of cortical lesion volume who presented with 4 foci of dura mater enhancement

Cortical LN and LV quantification was conducted using consensus-based criteria [31] via a combination of previously described and validated retrospective methods, including T2w-FLAIR-squared [32], artificial intelligence-double inversion recovery (AI-DIR) [33], and T1/T2 ratio [34]. The following raw images were used: 2D T2w, 3D T1w (IR-FSPGR), and 3D T2w-FLAIR. Raw images were pre-processed to remove bias field inhomogeneities [35], aligned to MNI space [36], resampled to 1 mm isotropic voxels, intensity standardized, and de-skulled [37]. T2w-FLAIR-squared images were created via multiplication of aligned 3D T2w-FLAIR and 2D T2w [32] and T1/T2 ratio images were created by dividing 3D T1w by the 2D T2w [34]. AI-DIR images were generated using a trained deep generative neural network, as described previously [33]. To provide additional context during lesion delineation, FastSurfer [27] was used to create cortical surface maps on 3T1w IR-FSPGR images. To further facilitate manual review and improve contrast, multi-modal cortical lesion enhanced (MMCLE) images were created via voxel-wise multiplication of AI-DIR and T2w-FLAIR-squared images [38]. A trained in-house semantic segmentation deep neural network was used as a preliminary step in lesion delineation to improve sensitivity and reliability [38]. In particular, cortical lesions were required to be brighter than normal gray matter (GM) on T2w-FLAIR-squared, AI-DIR, and MMCLE, or darker than normal GM on T1/T2 ratio. They were also required to be at least 3 voxels in size, and to not be commonly seen artifacts or traceable cortical vessels. To differentiate U-fiber lesions from true cortical involvement, we required each lesion to include at least two voxels fully within or directly touching the cortical surface. In the case of confluent lesions, only the portions of lesions within the 2 mm neighborhood of cortex were included, and only when the cortical lesion could be clearly visually separated from the rest of the confluent complex. We trained individual deep learning AI models on each contrast to compare detectability and reliability, as well as a final combined model to evaluate the potential value of simultaneous use of multiple contrasts. Blinded rater (*n* = 4) review of lesion detectability on each contrast, in 10 pwMS with various cortical lesion burden, was assessed by random presentation of lesions and matched non-lesions with random interleaving of contrasts. The reliability of the raters was compared against ground truth (consensus unblinded review of all contrasts simultaneously) and is reported as mean across raters using Cohen’s *κ*. The inter-rater comparison is reported as Fleiss’ *κ*. Lesions were identified by an expert neuroimager (AB, six years of experience) using a custom 3D Slicer module, based on simultaneous reference to all four lesion-sensitive contrasts (Fig. 1) [31].

### Meningeal enhancement patterns

**Fig. 2:**
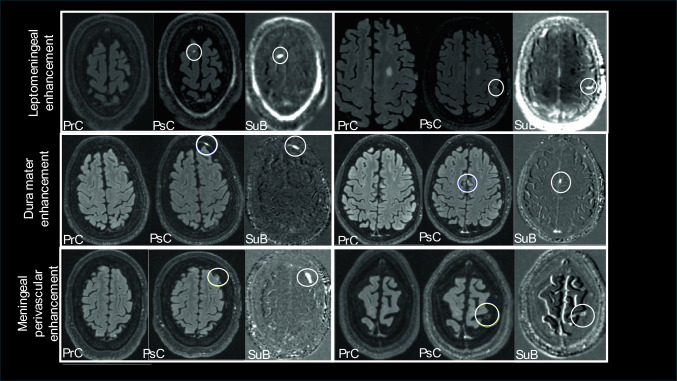
Types of meningeal and perivascular enhancement patterns in persons with multiple sclerosis (MS). From left to right are shown pre-contrast (PrC), post-contrast (PsC) and subtraction (SuB) images. Top row shows leptomeningeal enhancement (LME), middle row shows dura mater enhancement (DME) and bottom row shows meningeal perivascular enhancement (MPVE). White circles outline the LME, DME and MPVE. Top row shows LME in a 32-year-old female with relapsing–remitting (RR) MS on the left and a 45-year-old male with progressive MS on the right. Middle row shows DME in a 35-year-old male with RRMS on the left and a 48-year-old female with progressive MS on the right. Bottom row shows MPVE in a 40-year-old female with RRMS on the left and a 45-year-old male with progressive MS on the right

ME and MPVE patterns (Fig. 2) were evaluated using co-registered pre- and post-contrast 3D T2w FLAIR images and a subtraction map as aid to confirm the hyperintensities as previously described [8, 9, 13] and reported by others [15–17]. The subtraction map was generated by voxel-wise subtraction of the rigidly registered 3D T2w FLAIR pre- and post-contrast images. ME was defined as any meningeal enhancement, encompassing LME and DME. LME was identified as nodular or spread/fill enhancement within the subarachnoid space exceeding adjacent parenchymal signal intensity. DME was defined as enhancement along the dura mater outside of the subarachnoid space, and as enhancement localized to the falx cerebri. MPVE was defined as an enhancement within or surrounding the walls of meningeal cortical vessels and dural sinuses and was analyzed separately. In addition, the combined presence of ME/MPVE was evaluated to assess the coexistence of meningeal and vascular enhancement patterns. Pre- and post-contrast and subtraction images were reviewed in the sagittal, coronal, and axial planes using JIM software. Analyses were reviewed by two independent raters (FH with two years of experience, NB with 21 years of experience), and the expertise of a third experienced rater (RZ with 26 years of experience) was consulted in cases of uncertainty. Detection reproducibility of LME, DME, and MPVE was previously reported [9].

### Brain volume analysis

Whole brain, white matter (WM), GM, cortical and lateral ventricles volumes were determined on 3D T1w IR-FSPGR images using cross-sectional Structural Image Evaluation, using Normalization, of Atrophy (SIENAX) [39]. Similarly, deep GM volume and thalamic volume were also calculated on 3D T1w IR-FSPGR using FMRIB’s Integrated Registration and Segmentation Tool (FIRST) [40]. Lesion filling was performed prior to segmentation to minimize tissue misclassification [36].

### Data availability

The data that support the findings of this study are available on request from the corresponding author. The data are not publicly available due to privacy and ethical restrictions.

### Statistical analysis

Statistical analysis and visualizations were performed using SPSS version 29.0 (IBM, Armonk, NY, United States). Data distributions were assessed using visual inspection of the data and histograms, and model assumptions were checked using *Q*–*Q* plots. Categorical variables were compared using χ^2^ tests. Group comparisons were performed parametrically using Student’s *t* test (for normally distributed data) and non-parametrically using Mann–Whitney *U* test.

Analysis of covariance (ANCOVA), corrected for age, sex, disease duration, and disease subtype was performed to test differences between pwMS with presence and absence of ME, DME, LME and MPVE. *p* values and partial *η*^2^ effect size are shown. *p* values were Bonferroni-corrected for multiple comparisons.

To evaluate the potential influence of age, participants were stratified into three age groups: < 45 years, 45–55 years, and > 55 years. The presence and frequency of combined ME/MPVE, ME, DME, LME and MPVE, and cortical LN and LV were compared across age groups using the *χ*^2^ test (or Fisher’s exact test when appropriate) for categorical variables and one-way ANOVA for continuous variables.

Pearson correlation coefficients (*r*) were computed between ME, DME, LME and MPVE frequencies and other MRI outcomes and *p* values were Bonferroni-corrected.

Stepwise multiple linear regression was used to identify independent MRI predictors of cortical LN and LV. Age, sex, disease duration, disease subtype and DMT status (no DMT, low, medium and high efficacy) were forced into the first step to control for demographic effects and clinical confounders. All other MRI measures that showed significant Pearson correlations with DME, LME and MPVE were entered into a stepwise model, except T1-LV which was excluded due to high collinearity with T2-LV. Multicollinearity was checked (variance inflation factor < 3), and residuals were inspected for normality and homoscedasticity. Model performance was summarized with standardized *β*, adjusted *R*^2^, *F*-statistics, and *p* values were Bonferroni-corrected.

## Results

### Demographic and clinical characteristics

Table 1 and Supplement Table 2 show demographic and clinical characteristics of the included study cohort.

**Table 1:** Demographic and clinical characteristics in people with multiple sclerosis, according to the presence of meningeal and dura mater enhancement

Variable	Total MS (*n* = 214)	ME + (*n* = 121)	ME− (*n* = 93)	*p* value	DME + (*n* = 97)	DME*− *(*n* = 117)	*p *value
**Females, *n* (%)**	161 (75.2%)	91 (75.2%)	70 (75.3%)	1.000	72 (74.2)	113 (78.5%)	0.879
**Age at MRI exam, years, mean (SD)**	46.5 (11.4)	47.9 (11.7)	44.7 (10.9)	**0.041**	47.3 (11.8)	45.8 (11.2)	0.345
**Age at MS onset, years, mean (SD)**	31.6 (10.9)	32.8 (10.9)	30.1 (10.6)	*0.063*	31.5 (10.1)	31.7 (11.5)	0.396
**Disease duration, years, mean (SD)**	14.8 (9.4)	14.9 (8.9)	14.7 (10.0)	0.858	15.1 (9.2)	14.6 (9.5)	0.895
**Relapse rate in last past 24 months, mean (SD)**	0.4 (0.8)	0.4 (0.9)	0.4 (0.8)	0.867	0.4 (1.0)	0.4 (0.7)	0.635
**MS type, ** *n * (%)							
**RRMS**	173 (80.8)	96 (79.3)	77 (82.8)	0.601	74 (76.3)	99 (84.6)	0.162
**PMS**	41 (19.2)	25 (20.7)	15 (17.2)		23 (23.7	18 (15,4)	
**EDSS score, median (IQR)**	2.5 (2.0–3.5)	3.0 (2.0–4.0)	2.5 (1.5–3.5)	**0.022**	3.0 (2.0–4.0)	2.5 (1.5–3.5)	*0.064*
**DMT**, *n* (%)							
Anti-CD20	12 (5.6)	7 (5.8)	5 (5.4)	0.518	3 (3.1)	9 (7.7)	0.467
Oral therapies	48 (22.4)	23 (19.0)	25 (26.9)		19 (19.6)	29 (24.8)	
Interferon-beta	44 (20.6)	27 (22.3)	17 (18.3)		22 (22.7)	22 (18.8)	
Glatiramer acetate	31 (14.5)	14 (11.6)	17 (18.3)		11 (11.3)	20 (17.1)	
Natalizumab	30 (14.0)	18 (14.9)	12 (12.9)		15 (15.5)	15 (12.8)	
Other DMT	6 (2.8)	4 (3.3)	2 (2.2)		3 (3.1)	3 (2.6)	
No DMT	43 (20.1)	28 (23.1)	15 (16.1)		24 (24.7)	19 (16.2)	

MS, multiple sclerosis; ME, meningeal enhancement; DME, dura mater enhancement; +, positive; −, negative; SD, standard deviation; RRMS, relapsing, remitting MS; PMS, progressive MS; EDSS, Expanded Disability Status Scale; IQR, interquartile range; DMT, disease-modifying therapy

Interferon-beta therapies include intramuscular and subcutaneous interferon-beta 1a. Oral therapies include teriflunomide, dimethyl fumarate, fingolimod and diroximel fumarate. Anti-CD-20 therapies include ocrelizumab and rituximab. Other therapies include azathioprine, intravenous immunoglobulin, mitoxantrone and methylprednisolone

*χ*^2^ test, Student’s *t* test and Mann–Whitney *U* test were used to test difference between the groups. In bold are shown significant *p* values < 0.05 and in italic *p* values < 0.01

Of the 214 pwMS selected for the study, 142 (66.4%) showed combined presence of ME/MPVE, 121 (56.5%) ME, 97 (45.3%) DME, 48 (22.4%) LME, and 46 (21.5%) MPVE (Table 2). Subgroup analyses revealed that LME + pwMS were older (51.9 vs. 44.9 years, *p* < 0.001) and had older age at disease onset (36.3 vs. 30.2 years, *p* < 0.001) compared to LME− pwMS. MPVE + pwMS were older than MPVE- pwMS (49.8 vs. 45.6 years, *p* = 0.028). Age-stratified analyses (< 45, 45–55, > 55 years) showed similar presence of DME and MPVE patterns across the three age groups. However, LME was more frequent in older age groups (*p* = 0.002), and there was a statistical trend toward higher prevalence of overall ME (*p* = 0.078) and combined ME/MPVE (*p* = 0.063) patterns (Table 2). No differences in cortical lesion burden were found across age groups (Table 2). There were no significant differences in the prevalence of ME, DME, LME or MPVE between RRMS and PMS groups (Table 1 and Supplement Table 2).

**Table 2:** Distribution of meningeal enhancement patterns and cortical lesions across age groups in people with multiple sclerosis

Variable	All age groups (*n* = 214)	> 45 years old (*n* = 91)	45–55 years old (*n* = 60)	> 55 years old (*n* = 63)	*p * value
*Presence*					
DME +	97 (56.5)	39 (42.9)	26 (43.3)	32 (50.8)	0.583
LME +	48 (22.4)	11 (12.1)	14 (23.3)	18 (28.6)	**0.002**
MPVE +	46 (21.5)	14 (15.4)	14 (23.3)	18 (28.6)	0.135
ME +	121 (56.5)	46 (50.5)	32 (53.3)	43 (68.3)	*0.078*
Combined ME + and MPVE +	142 (66.4)	53 (58.2)	41 (68.3)	48 (76.2)	*0.063*
*Frequency*					
DME frequency	0.8 (1.1)	0.8 (1.2)	0.73 (1.1)	0.8 (1)	0.951
LME frequency	0.36 (0.8)	0.15 (0.4)	0.4 (0.8)	0.6 (1.1)	**0.002**
MPVE frequency	0.27 (0.6)	0.22 (0.4)	0.28 (0.6)	0.3 (0.6)	0.514
ME frequency	1.2 (1.4)	1 (1.3)	1.2 (1.5)	1.47 (1.5)	0.143
Combined ME/MPVE frequency	1.4 (1.6)	1.1 (1.5)	1.4 (1.5)	1.7 (1.7)	*0.083*
*Cortical lesions*					
Cortical LN	7 (9.3)	7.5 (10.4)	6.9 (6.8)	6.6 (9.6)	0.809
Cortical LV	270.6 (421.8)	270.1 (418.9)	267.4 (340.4)	274.4 (496.2)	0.996

MS, multiple sclerosis; DME, dura mater enhancement; LME, leptomeningeal enhancement; MPVE, meningeal perivascular enhancement; ME, meningeal enhancement; +, positive; SD, standard deviation; LN, lesion number; LV, lesion volume.

All measures for DME + , LME + , MPVE + , ME + and combined ME/MPVE people with MS are shown as *n* and (%). Enhancement frequencies and cortical lesion burden are reported as mean (standard deviation). Cortical LV is reported in millimeter cubes

χ^2^ test (or Fisher’s exact test when appropriate) for categorical and analysis of variance (ANOVA) for continuous variables were used to test difference between the groups. In bold are shown significant *p* values < 0.05 and in italic *p* values < 0.01

In pwMS, the mean numbers (SD) of enhancement foci were: combined ME/MPVE 1.4 (1.6), ME 1.2 (1.4), LME 0.36 (0.8), DME 0.8 (1.1), and MPVE 0.27 (0.6) (Table 2). Age-stratified analyses (< 45, 45–55, > 55 years) showed similar frequencies of combined ME/MPVE, ME, DME and MPVE patterns and cortical LN and LV across age groups. However, LME was significantly more frequent in older age groups (*p* = 0.002), with a trend toward higher combined ME/MPVE frequency (*p* = 0.083) (Table 2). A significantly higher number of enhancement foci was detected in the PMS group compared to the RRMS one for ME (1.8 vs 1.1, *p* < 0.001) and DME (1.3 vs. 0.6, *p* < 0.001), but not for LME (0.4 vs. 0.3, *p* = 0.192) or MPVE (0.29 vs. 0.27, *p* = 0.454).

Sex distribution did not differ between enhancement-positive and negative groups (all *p* > 0.3). ME + pwMS had a slightly higher mean age compared with ME- pwMS (47.9 vs. 44.7 years, *p* = 0.041), while disease duration and relapse rate did not differ. EDSS score was higher in ME + than ME− pwMS (median 3.0 vs. 2.5, *p* = 0.022). DME + patients showed a similar but non-significant trend (*p* = 0.064).

LME + pwMS had a slightly lower relapse rate in the preceding 2 years (0.2 vs. 0.5, *p* = 0.023). No significant differences in DMT exposure were detected across enhancement subgroups.

### Reliability for detection of cortical lesions

The highest Cohen’s *κ* was detected for MMCLE contrast (*κ* = 0.78) followed by T1/T2 ratio (*κ* = 0.60), T2w-FLAIR-squared (*κ* = 0.58), AI-DIR (*κ* = 0.56), T2-WI (*κ* = 0.35) and T2w-FLAIR (*κ* = 0.13). The highest inter-rater *κ* was detected for MMCLE contrast (*κ* = 0.73) followed by AI-DIR (*κ* = 0.68), T2w-FLAIR-squared (*κ* = 0.65), T1/T2 ratio (*κ* = 0.47), T2w-FLAIR (*κ* = 0.41) and T2-WI (*κ* = 0.36.

### Comparisons between groups split by enhancement patterns

MRI outcome comparisons in pwMS, according to the presence of enhancement patterns, are shown in Tables 3 (ME and DME) and 4 (LME and MPVE).

**Table 3:** MRI outcomes comparisons in persons with multiple sclerosis, according to the presence of meningeal and dural mater enhancement

Variable	ME + (*n* = 121)	ME− (*n* = 93)	p*/p** values	Partial *η*^2^	DME + (*n* = 97)	DME− (*n* = 117)	p*/p** values	Partial *η*^2^
CE-LN	0.4 (1.7)	0.2 (0.5)	0.105/1.000	0.013	0.6 (0.9)	0.3 (1.6)	0.841/1.000	0.000
CE-LV	51.9 (225.5)	40.1 (161.3)	0.560/1.000	0.002	40.7 (120.5)	51.8 (247.4)	0.801/1.000	0.000
T2-LV	13.2 (13.7)	7.9 (9.7)	**0.003/0.045**	**0.04**	13.5 (13.3)	8.7 (11.2)	*0.007/0.09*	*0.035*
T1-LV	2.5 (3.7)	1.3 (1.8)	0.008/0.106	0.033	2.5 (3.2)	1.5 (2.7)	0.023/0.296	0.025
CLN	8.8 (10.6)	4.9 (6.6)	**0.001/0.013**	**0.051**	9.6 (11.7)	4.9 (6.2)	**< 0.001/0.003**	**0.062**
CLV	343.5 (486.8)	178.9 (299)	**0.004/0.048**	**0.04**	374.1 (526.9)	187.6 (288.4)	**0.001/0.019**	**0.048**
WBV	1472 (101)	1505.1 (99.8)	0.075/0.975	0.015	1477.3 (102.6)	1493.4 (100.6)	0.447/1.000	0.003
WMV	681.4 (41.9)	690.7 (39.6)	0.167/1.000	0.009	683.4 (41.9)	687.1 (40.5)	0.699/1.000	0.001
GMV	790.6 (74.4)	814.3 (73.2)	0.102/1.000	0.013	793.9 (76.6)	806.8 (72.7)	0.409/1.000	0.003
CGMV	617.5 (54.9)	633.7 (57)	0.176/1.000	0.009	620 (56.2)	628.4 (56.4)	0.533/1.0000	0.002
LVV	54.4 (24.2)	44.2 (20.4)	*0.005/0.06*	*0.038*	54.8 (23.7)	46 (21.9)	0.011/0.138	0.031
DGMV	56 (8.6)	59.2 (7.4)	0.014/0.176	0.029	55.7 (8.4)	58.7 (7.9)	0.02/0.255	0.026
TV	18.1 (2.7)	19.2 (2.6)	0.011/0.141	0.031	18.1 (2.7)	19 (2.7)	0.03/0.385	0.023

MS, multiple sclerosis; ME, meningeal enhancement; DME, dura mater enhancement; +, positive; −, negative; SD, standard deviation; LN, lesion number; LV, lesion volume; CE, contrast-enhancing; C, cortical; WBV, whole brain volume; WMV, white matter volume; GMV, gray matter volume; CGMV, cortical gray matter volume; LVV, lateral ventricle volume; DGMV, deep gray matter volume; TV, thalamic volume

All measures are reported as mean (standard deviation). CE-LV and CLV are reported in millimeter cubes. All other volumes are reported in milliliters. Analysis of covariance (ANCOVA), adjusted for age, sex, disease duration and disease subtype was used to explore differences between the groups. *p* values and Partial *η*^2^ effect size are shown between ME + and ME− and DME + and DME− groups. Both *uncorrected and **corrected *p* values are reported; results were considered significant at *p* < 0.05 after Bonferroni correction. In bold are shown significant *p* values < 0.05 and in italic *p* values < 0.01

**Table 4:** MRI outcomes comparisons in persons with multiple sclerosis, according to the presence of leptomeningeal and vessel wall enhancement

Variable	LME + (*n* = 48)	LME − (*n* = 166)	p*/p** values	Partial *η*^2^	MPVE + (*n* = 46)	MPVE − (*n* = 168)	p*/p** values	Partial *η*^2^
CE-LN	0.7 (2.5)	0.3 (0.6)	0.008/0.106	0.033	0.2 (0.6)	0.4 (1.5)	0.748/1.000	0.001
CE-LV	80.8 (336.2)	36.8 (136.6)	0.083/1.000	0.014	31.4 (118.8)	51 (217.1)	0.725/1.000	0.001
T2-LV	13.6 (16.2)	10.1 (11)	0.270/1.000	0.006	13.4 (14.4)	10.2 (11.7)	0.267/1.000	0.006
T1-LV	2.7 (4.4)	1.7 (2.4)	0.147/1.000	0.01	2.7 (4)	1.7 (2.6)	0.119/1.000	0.012
CLN	7 (9)	7 (9.5)	0.797/1.000	0.000	6.3 (8.3)	7.3 (9.6)	0.588/1.000	0.001
CLV	294.4 (476.8)	265 (406.7)	0.650/1.000	0.000	262.7 (398.7)	274.1 (430)	0.826/1.000	0.000
WBV	1452.8 (94.7)	1496.2 (101.7)	0.183/1.000	0.001	1476.5 (100.3)	1489.2 (102)	0.819/0.917	0.000
WMV	675.7 (38.7)	688.3 (41.4)	0.178/1.000	0.009	677.1 (36.3)	687.8 (42.1)	0.254/1.000	0.006
GMV	777.1 (69.9)	807.9 (74.7)	0.304/1.000	0.005	799.4 (77.3)	801.4 (74.1)	0.305/1.000	0.005
CGMV	606.5 (51.5)	629.9 (56.6)	0.326/1.000	0.005	623 (60.3)	625.1 (55.3)	0.314/1.000	0.005
LVV	59.3 (27.8)	47.2 (20.9)	0.019/0.248	0.026	55.2 (25.7)	48.5 (22.2)	0.258/1.000	0.006
DGMV	56.4 (87)	57.7 (8.2)	0.919/1.000	0.000	56.9 (86)	57.5 (81.2)	0.846/1.000	0.000
TV	18 (2.7)	18.8 (2.7)	0.255/1.000	0.006	18.4 (2.8)	18.7 (2.7)	0.921/1.000	0.000

MS, multiple sclerosis; LME, leptomeningeal enhancement; MPVE, meningeal perivascular enhancement; +, positive; −, negative; SD, standard deviation; LN, lesion number; LV, lesion volume; CE, contrast-enhancing; C, cortical; WBV, whole brain volume; WMV, white matter volume; GMV, gray matter volume; CGMV, cortical gray matter volume; LVV, lateral ventricle volume; DGMV, deep gray matter volume; TV, thalamic volume

All measures are reported as mean (standard deviation). CE-LV and CLV are reported in millimeter cubes. All other volumes are reported in milliliters

Analysis of covariance (ANCOVA), adjusted for age, sex, disease duration and disease subtype was used to explore differences between the groups. *p* values and Partial *η*^2^ effect size are shown between LME + and LME− and MPVE + and MPVE- groups. Both *uncorrected and **corrected *p* values are reported; results were considered significant at *p* < 0.05 after Bonferroni correction

CE lesions were present in 33 of 214 (15.4%) patients. Among these, 21 (63.6%) showed the presence of combined ME/MPVE, while the ME/MPVE frequency in 181 pwMS without CE lesions was similar (66.8%). No significant correlations were observed between meningeal enhancement patterns and CE lesion number or volume.

After adjusting for age, sex, disease duration, and disease subtype, as well as correcting for multiple comparisons, ANCOVA showed that ME + pwMS had significantly higher cortical LN (8.8 vs. 4.9, *p*_corr_ = 0.013), T2-LV (13.2 vs. 7.9 mL, *p*_corr_ = 0.045), and cortical LV (343.5 vs. 178.9 mm^3^, *p*_corr_ = 0.048). DME + pwMS showed even stronger differentiation with respect to the cortical lesion pathology: cortical LN (9.6 vs. 4.9, *p*_corr_ = 0.003) and cortical LV (374.1 vs. 187.6 mm^3^, *p*_corr_ = 0.019). DME + patients also exhibited a trend toward greater T2-LV (*p*_corr_ = 0.09).

In contrast, LME and MPVE groups showed no significant differences in cortical or WM lesion metrics after correction for multiple comparison. LME + patients had nominally higher CE-LN (*p*_uncorr_ = 0.008) and greater LVV (*p*_uncorr_ = 0.019), but these associations did not survive correction (*p*_corr_ = 0.106 and 0.248, respectively). MPVE presence was not related to any lesion or volumetric outcome.

### Correlation analysis

Across all participants, ME and DME frequencies correlated positively with lesion burden and inversely with brain volumes (Table 5). ME frequency correlated with cortical LN (*r* = 0.477, *p* < 0.001), cortical LV (*r* = 0.460, *p* < 0.001) and T2-LV (*r* = 0.425, *p* < 0.001). DME frequency showed even stronger associations with cortical LN (*r* = 0.586, *p* < 0.001) and cortical LV (*r* = 0.555, *p* < 0.001) (Fig. 1). LME frequency demonstrated weaker but significant correlations with T2-LV, T1-LV, and lateral ventricle volume (*p* < 0.001), while MPVE frequency did not correlate significantly with any MRI outcomes. These correlations remained significant after correction for multiple comparisons.

**Table 5:** Pearson correlation coefficients between frequency of meningeal, dura mater, leptomeningeal and perivascular enhancement foci and MRI outcomes in persons with multiple sclerosis

Variable	ME frequency		DME frequency		LME frequency		MPVE frequency	
	*r*	*p*	*r*	*p*	*r*	*p*	*r*	*p*
CE-LN	0.051	0.462	0.019	0.788	0.077	0.266	− 0.008	0.906
CE-LV	0.009	0.895	− 0.023	0.738	0.065	0.342	− 0.026	0.703
T2-LV	**0.425**	** < 0.001**	**0.343**	** < 0.001**	**0.247**	** < 0.001**	0.142	0.038
T1-LV	**0.335**	** < 0.001**	**0.223**	**0.001**	**0.298**	** < 0.001**	0.096	0.164
CLN	**0.477**	** < 0.001**	**0.586**	** < 0.001**	0.021	0.758	0.063	0.358
CLV	**0.460**	** < 0.001**	**0.555**	** < 0.001**	0.024	0.729	0.091	0.186
WBV	− **0.296**	** < 0.001**	− 0.199	0.003	− 0.171	0.012	− 0.074	0.278
WMV	− **0.233**	** < 0.001**	− 0.132	0.053	− 0.129	0.059	− 0.129	0.060
GMV	− **0.274**	** < 0.001**	− 0.198	0.004	− 0.162	0.018	− 0.031	0.657
CGMV	− **0.261**	** < 0.001**	− 0.186	0.006	− 0.150	0.029	− 0.036	0.596
LVV	**0.371**	** < 0.001**	**0.280**	** < 0.001**	**0.244**	** < 0.001**	0.109	0.111
DGMV	**− 0.380**	** < 0.001**	− **0.334**	** < 0.001**	− 0.152	0.027	− 0.048	0.487
TV	**− 0.382**	** < 0.001**	− **0.311**	** < 0.001**	− 0.187	0.006	− 0.078	0.255

MS, multiple sclerosis; ME, meningeal enhancement; DME, dura mater enhancement; LME, leptomeningeal enhancement; MPVE, meningeal perivascular enhancement; +, positive; −, negative; LN, lesion number; LV, lesion volume; CE, contrast-enhancing; C, cortical; WBV, whole brain volume; WMV, white matter volume; GMV, gray matter volume; CGMV, cortical gray matter volume; LVV, lateral ventricle volume; DGMV, deep gray matter volume; TV, thalamic volume.

Pearson correlations were computed between ME, DME, LME and MPVE frequencies and other MRI measures. In bold are shown *p* values < 0.05 that remained significant after Bonferroni correction

### Regression analysis

Stepwise multiple linear regression identified DME frequency as independent predictor of both cortical LN and LV after controlling for age, sex, disease duration, disease subtype and DMT status (Table 6).

**Table 6:** Stepwise multiple linear regression predicting cortical lesion number and volume in persons with multiple sclerosis

	Standardized *β*	*t*	*p *value	Adjusted Δ*R*^2^	*p *value of the model
*Cortical lesion number*					
Demographic and clinical variables					
Sex	− 0.06	− 0.6	0.547	0.06	**0.179**
Disease duration	− 0.05	− 0.40	0.687		
Disease subtype	− 0.05	− 0.4	0.703		
DMT	0.08	0.8	0.423		
Age	− 0.22	− 0.19	0.503		
MRI outcomes					
T2-LV	0.52	4.4	**< 0.001**	0.39	** < 0.001**
DME frequency	0.45	3.9	**< 0.001**	0.54	
*Cortical lesion volume*					
Demographic and clinical variables					
Sex	− 0.46	− 0.46	0.687	0.01	**0.364**
Disease subtype	− 0.07	1.02	0.308		
Disease duration	− 0.38	− 0.43	0.666		
DMT	0.06	0.48	0.629		
Age	− 0.43	− 0.35	0.730		
MRI outcomes					
T2-LV	0.49	3.78	**< 0.001**	0.3	**< 0.001**
DME frequency	0.41	3.17	**0.003**	0.42	

MS, multiple sclerosis; DME, dura mater enhancement; LV, lesion volume; DMT, disease-modifying therapy

Low efficacy DMTs were interferon-beta, glatiramer acetate and other DMT; medium efficacy were oral therapies, and high efficacy were anti-CD20 and natalizumab)

Stepwise multiple linear regression was used to predict cortical lesion number and volume. Age, sex, disease duration, disease subtype and DMT status (no DMT, low, medium and high efficacy) were forced into the model; MRI variables that survived Bonferroni correction in Pearson analysis were entered stepwise. Standardized *β*, *t*, *p* value, adjusted *R*^2^, and *F* model change statistics are shown for retained predictors. In bold are shown *p* values that remained significant after Bonferroni correction

The overall models were significant for both outcomes (cortical LN*:* *F* = 9.3, *R*^2^ = 0.54; cortical LV: *F* = 6.2, *R*^2^ = 0.42; both *p* < 0.001). Adding MRI metrics to demographic covariates increased explained variance from 6 to 54% (LN) and 1% to 42% (LV). Within the final models, DME frequency contributed 13–16% of unique variance, with additional independent effect from T2-LV; all MRI predictors remained significant after correction for multiple comparisons.

## Discussion

This study demonstrates that DME is a common imaging finding in pwMS and is associated with greater cortical lesion burden on 3T MRI. Importantly, these results describe an imaging association and do not establish a causal, inflammatory, or biological mechanism linking DME to cortical demyelination. The observed relationships should therefore be interpreted within the known age-related changes in meningeal permeability. DME frequency correlated significantly with both cortical LN and LV, while LME or MPVE showed no meaningful relationship with cortical lesion burden or other MRI measures. Regression analysis confirmed DME as an independent predictor of cortical lesion pathology, explaining 13–16% of variance beyond demographic, clinical and conventional MRI measures. DMT status was included as a covariate in regression models and did not change the association between DME and cortical lesion burden. This suggests that the observed relationship is unlikely to be driven by differences in DMT exposure.

Previous studies showed that increased LME is associated with older age [2, 4, 5, 7, 11, 16–18, 20]. Age-stratified analyses in the present study showed similar frequencies of DME and MPVE across age groups, with no differences in cortical lesion burden. In contrast, in line with literature [2, 4, 5, 7, 11, 16–18, 20], LME was significantly more frequent in older age groups, with a trend toward higher combined ME/MPVE frequency. These findings suggest that LME may increase with age or disease duration, whereas DME appears less age dependent. Importantly, the association between DME and cortical lesion burden observed in the primary analyses was not driven by age-related effects.

Although ME/MPVE were observed in patients with CE lesions, there was no difference between CE + and CE − groups, and their presence was not associated with CE lesion number or volume. This suggests that meningeal enhancement does not directly reflect the burden of focal blood–brain barrier disruption.

Recent evidence has redefined the dura mater as an active interface in neuro-immune and fluid-exchange processes rather than a passive structural barrier of the CNS. In a seminal study, Smyth et al. [21] identified arachnoid cuff exit (ACE) points, discrete anatomical discontinuities where bridging veins traverse the arachnoid barrier, thereby creating direct conduits between the dura mater and the subarachnoid space. These structures enable CSF and solute exchange under physiological conditions and may facilitate immune cell trafficking from the dura into subarachnoid compartments in neuro-inflammatory states. This discovery provides a potential anatomical framework through which dural and cortical compartments may interact although the present study does not directly test these mechanisms. DME observed on post-contrast MRI may therefore reflect regions of altered regulation at the dura–cortex interface, permitting bidirectional exchange of fluid and solutes. In pwMS, such dysregulated communication across ACE interfaces could influence cortical pathology through altered meningeal–cortical interactions. Notably, the dural vasculature is fenestrated and lacks the tight blood–brain barrier of pial vessels, potentially increasing its sensitivity to systemic signals.

Our analyses demonstrated that DME was associated with cortical LN and LV but showed no robust associations with other conventional MRI measures. A nominal difference in T2-LV between DME + and DME pwMS was observed prior to correction for multiple comparisons but did not persist after adjustment. Overall, this pattern suggests that DME is not simply a surrogate of global lesion burden or neurodegeneration but rather represents a spatially localized imaging finding preferentially associated with CL burden. The presence of DME does not necessarily indicate pathological inflammation and may instead reflect altered contrast distribution or vascular permeability related to cumulative cortical lesion burden rather than a disease-specific inflammatory process.

In contrast, LME was poorly correlated with cortical lesion burden and showed a minimal association with other MRI metrics in this cohort. To date, no 3T MRI studies have systematically evaluated the relationship between LME and quantitative cortical lesion metrics. While 7T studies [2, 19, 20] have suggested stronger spatial and quantitative associations between LME and cortical pathology, the lower field strength and limited sensitivity of 3T MRI may reduce the ability to detect subtle intracortical and subpial inflammatory changes. These methodological differences likely explain the weaker correlations observed in our analysis.

Our evaluation of MPVE also did not reveal meaningful associations with cortical or other MRI measures. This absence of correlation suggests that the type of enhancement observed here likely represents perivascular changes confined to the meningeal vessel walls rather than the deeper paravascular pathways implicated in immune cell trafficking [26]. While perivascular inflammation may occur as part of the broader meningeal inflammatory milieu, it might not directly contribute to cortical lesion formation. By contrast, paravascular inflammation, described in recent work showing that CSF-borne macromolecules can move from periarterial to perivenous spaces through specialized arteriovenous perivascular overlaps, may be more relevant to MS pathophysiology [26]. These paravascular conduits facilitate macromolecular clearance, fluid shunting, and immune surveillance, functions not captured by superficial perivascular enhancement detectable at 3T MRI.

Because ME represents the combined presence of DME and LME, this metric captures the overlap between pachymeningeal and leptomeningeal enhancement patterns. The additional evaluation of combined ME/MPVE further allowed assessment of the coexistence of meningeal and vascular inflammatory features. Despite this overlap, DME maintained its association with cortical lesion burden, supporting the interpretation that pachymeningeal inflammation may represent a distinct component of meningeal pathology in MS.

Several limitations should be acknowledged. First, this study was conducted at 3T, which limits spatial resolution compared with 7T MRI and almost certainly reduces sensitivity to detection of intracortical or subpial lesions. Second, we did not differentiate between leukocortical, intracortical, and subpial lesion subtypes, which could have otherwise helped further refine the interpretation of the relationship between DME and cortical pathology. Third, we did not perform regional or voxel-wise analyses to directly compare the spatial distribution of DME and cortical lesions, which would help establish their topographical relationship. Although cortical lesions in the present study were assessed by a single experienced reader, the multi-modal detection pipeline used underwent blinded validation with four independent raters demonstrating good inter-rater agreement and lesion detectability for the MMCLE contrast. Finally, this was a cross-sectional analysis; therefore, longitudinal studies are needed to determine whether the presence or persistence of DME predicts subsequent cortical lesion formation or clinical progression.

In conclusion, DME on 3T MRI is associated with increased cortical lesion burden in pwMS. These findings highlight a robust imaging correlation but do not imply a direct biological or inflammatory mechanism. Future studies incorporating longitudinal imaging, higher field strengths, histopathology, and physiological measures of meningeal permeability and contrast kinetics will be required to clarify the clinical and biological significance of DME in pwMS.

## Supplementary Information

Below is the link to the electronic supplementary material.

Supplementary file1 (DOCX 22 KB)

Supplementary file2 (DOCX 15 KB)
