# Supplementary material for: Dura mater enhancement on 3T MRI is associated with cortical lesion burden in multiple sclerosis
Source: J Neurol. 2026 Apr 6;273(4):251. doi: 10.1007/s00415-026-13781-6 (PMC13053467; doi:10.1007/s00415-026-13781-6)
Supplement: Supplementary file 1 — Supplementary file1 (DOCX 22 KB) [file 415_2026_13781_MOESM1_ESM.docx]

**Supplement Table 1.** Specification of MRI acquisition sequences.

|  | **3DT1w IR-FSPGR** | **3D T2w FLAIR** | **3D T2w FLAIR +Gd** | **3D T1w SE** | **3D T1w SE +Gd** | **2D T2wSE** |
| --- | --- | --- | --- | --- | --- | --- |
| **Series acquisition time (minutes:seconds)** | 5:11 | 7:51 | 7:51 | 5:12 | 5:12 | 3:52 |
| **Orientation** | Sagittal | Sagittal | Sagittal | Sagittal | Sagittal | Axial |
| **Alignment** | Parallel to the sub-callosal line | Parallel to the sub-callosal line | Parallel to the sub-callosal line | Parallel to the sub-callosal line | Parallel to the sub-callosal line | Parallel to the sub-callosal line |
| **Phase direction** | ROW | Row | Row | Row | Row | Row |
| **Voxel size** | 1.0 x 1.0 x 1.2 mm^3^ | 1.4 x 1.0 x 1.0  mm^3^ | 1.4 x 1.0 x 1.0 mm^3^ | 0.9 x 0.9 x 10.0 mm^3^ | 0.9 x 0.9 x 10.0 mm^3^ | 0.94 x 0.94 x 3.0 mm^3^ |
| **Repetition time (TR; ms)** | 5.5 | 9000 | 9000 | 534 | 534 | 4350 |
| **Echo time (TE; ms)** | 2.2 | 107 | 107 | 12.8 | 12.8 | 104.96 |
| **Inversion time (TI; ms)** | 600 | 2405 | 2405 | NA | NA | NA |
| **Flip angle** | 8 | 90 | 90 | 90 | 90 | 90 |
| **Echo train length** | 1 | 192 | 192 | 24 | 24 | 24 |
| **Bandwidth** | 244.141 | 325.508 | 325.508 | 390.625 | 390.625 | 195.312 |
| **Percent phase FOV** | 100 | 100 | 100 | 100 | 100 | 100 |
| **Matrix size** | 192x192 | 224x224 | 224x224 | 256x256 | 256x256 | 320x224 |
| ***n* slices** | 156 | 140 | 140 | 190 | 190 | 44 |
| **Slice thickness (mm)** | 1.2 | 1.2 | 1.2 | 0.9 | 0.9 | 3 |
| **Spacing between slices (mm)** | 1.2 | 1.2 | 1.2 | 0.9 | 0.9 | 3 |
| **Contrast enhancement** | NA | NA | Gadolinium (0.1 mMol/kg), image acquired 10 minutes post-contrast administration | NA | Gadolinium (0.1 mMol/kg), image acquired 5 minutes post-contrast administration | NA |

MR-magnetic resonance; IR-FSPGR-inversion recovery - fast spoiled gradient echo; 3D-Three-dimensional; 2D-Two-dimensional; w-weighted FLAIR-fluid-attenuated inversion recovery; +Gd-gadolinium contrast; SE-spin-echo; FOV: field of view.

**Supplement Table 2.** Demographic and clinical characteristics in persons with multiple sclerosis, according to the presence of leptomeningeal and perivascular enhancement.

| **Variable** | **LME+**  **(n=48)** | **LME-**  **(n=166)** | **p**  **value** | **MPVE+**  **(n=46)** | **MPVE- (n=168)** | **p**  **value** |
| --- | --- | --- | --- | --- | --- | --- |
| **Females, n (%)** | 38 (79.2) | 123 (74.1) | 0.571 | 36 (78.3) | 125 (74.4) | 0.701 |
| **Age at MRI exam, years, mean (SD)** | 51.9 (11.0) | 44.9 (11.1) | **<0.001** | 49.8 (11.0) | 45.6 (11.4) | **0.028** |
| **Age at MS onset, years, mean (SD)** | 36.3 (11.0) | 30.2 (10.5) | **<0.001** | 33.7 (11.7) | 31.1 (10.6) | 0.179 |
| **Disease duration, years, mean (SD)** | 15.5 (9.8) | 14.6 (9.3) | 0.574 | 16.1 (8.8) | 14.4 (9.5) | 0.268 |
| **Relapse rate in last past 24 months, mean (SD)** | 0.2 (0.5) | 0.5 (0.9) | **0.023** | 0.2 (0.6) | 0.5 (0.9) | *0.064* |
| **MS type, n (%)**  **RRMS**  **PMS** | 38 (79.2)  10 (20.8) | 135 (81.3)  31(18.7) | 0.835 | 38 (82.6)  8 (17.4) | 135 (80.4)  33 (19.6) | 0.837 |
| **EDSS score, median (IQR)** | 3.0 (2.5-4.0) | 2.5 (1.5-3.5) | **0.011** | 3.0 (2.0-3.5) | 2.5 (1.5-3.5) | 0.684 |
| **DMT, n (%)**  Anti-CD20  Oral therapies  Interferon-beta  Glatiramer acetate  Natalizumab  Other DMT  No DMT | 4 (8.3)  9 (18.8)  13 (27.1)  8 (16.7)  6 (12.5)  1 (2.1)  7 (14.6) | 8 (4.8)  39 (23.5)  31 (18.7)  23 (13.9)  24 (14.5)  5 (3.0)  36 (21.7) | 0.834 | 4 (8.7)  7 (15.2)  8 (17.4)  9 (19.6)  7 (15.2)  1 (2.2)  10 (21.7) | 8 (4.8)  41 (24.4)  36 (21.4)  22 (13.1)  23 (13.7)  5 (3.0)  33 (19.6) | 0.897 |

**Legend:** MS-multiple sclerosis; LME-leptomeningeal enhancement; MPVE-meningeal perivascular enhancement; + positive; - negative; SD-standard deviation; RRMS-relapsing-remitting MS; PMS-progressive MS; EDSS-Expanded Disability Status Scale; IQR-interquartile range; DMT-disease-modifying therapy.

Interferon-beta therapies include intramuscular and subcutaneous interferon-beta 1a. Oral therapies include teriflunomide, dimethyl-fumarate, fingolimod and diroximel fumarate. Anti-CD-20 therapies include ocrelizumab and rituximab. Other therapies include azathioprine, intravenous immunoglobulin, mitoxantrone and methylprednisolone.

Chi-square test, Student’s t-test and Mann-Whitney U test were used to test difference between the groups. In bold are shown significant p values <0.05 and in italic p values <0.01.
