# Supplementary material for: Dura mater enhancement on 3T MRI is associated with cortical lesion burden in multiple sclerosis
Source: J Neurol. 2026 Apr 6;273(4):251. doi: 10.1007/s00415-026-13781-6 (PMC13053467; doi:10.1007/s00415-026-13781-6)
Supplement: Supplementary file 2 — Supplementary file2 (DOCX 15 KB) [file 415_2026_13781_MOESM2_ESM.docx]

**Supplement Figure.** A flow diagram summarizing the screening, inclusion, and exclusion of participants from the BUFFALO-MS database.

| BUFFALO-MS database pwMS (n = 3,863) |
| --- |

↓

| MRI scans performed in 2017–2018 (n = 2,735) |
| --- |

↓

| 3T MRI with required protocol (pre- and post-contrast 3D T2w FLAIR and conventional sequences) (n = 252) |
| --- |

↓

| Excluded (n = 38) • Missing clinical data (n = 30) • MRI outside 30‑day clinical window (n = 8) |
| --- |

↓

| Final study cohort included in analysis (n = 214) |
| --- |
